# Transcriptome dynamics uncovers long non-coding RNAs response to salinity stress in *Chenopodium quinoa*

**DOI:** 10.3389/fpls.2022.988845

**Published:** 2022-09-20

**Authors:** Chuping Luo, Bing He, Pibiao Shi, Jinlong Xi, Hongbing Gui, Bingwen Pang, Junjie Cheng, Fengqin Hu, Xi Chen, Yuanda Lv

**Affiliations:** ^1^School of Life Sciences and Food Engineering, Huaiyin Institute of Technology, Huaian, China; ^2^Excellence and Innovation Center, Jiangsu Academy of Agricultural Sciences, Nanjing, China; ^3^Zhejiang Institute of Standardization, Hangzhou, China; ^4^College of Animal Husbandry and Veterinary Medicine, Jiangsu Vocational College of Agriculture and Forestry, Jurong, China; ^5^School of Agronomy and Horticulture, Jiangsu Vocational College of Agriculture and Forestry, Jurong, China

**Keywords:** quinoa, LncRNA, salt stress, RNA-seq, gene co-expression

## Abstract

Chenopodium quinoa is a crop with outstanding tolerance to saline soil, but long non-coding RNAs (LncRNAs) expression profile driven by salt stress in quinoa has rarely been observed yet. Based on the high-quality quinoa reference genome and high-throughput RNA sequencing (RNA-seq), genome-wide identification of LncRNAs was performed, and their dynamic response under salt stress was then investigated. In total, 153,751 high-confidence LncRNAs were discovered and dispersed intensively in chromosomes. Expression profile analysis demonstrated significant differences between LncRNAs and coding RNAs. Under salt stress conditions, 4,460 differentially expressed LncRNAs were discovered, of which only 54 were differentially expressed at all the stress time points. Besides, strongly significantly correlation was observed between salt-responsive LncRNAs and their closest neighboring genes (*r* = 0.346, *p*-value < 2.2e-16). Furthermore, a weighted co-expression network was then constructed to infer the potential biological functions of LncRNAs. Seven modules were significantly correlated with salt treatments, resulting in 210 hub genes, including 22 transcription factors and 70 LncRNAs. These results indicated that LncRNAs might interact with transcription factors to respond to salinity stress. Gene ontology enrichment of the coding genes of these modules showed that they were highly related to regulating metabolic processes, biological regulation and response to stress. This study is the genome-wide analysis of the LncRNAs responding to salt stress in quinoa. The findings will provide a solid framework for further functional research of salt responsive LncRNAs, contributing to quinoa genetic improvement.

## Introduction

Quinoa (*Chenopodium quinoa* Willd.), an ancient annual dicotyledonous crop in the *Chenopodiaceae* family with approximately 7,000 years of cultivation, originated from the Andes mountains of South America (Jacobsen et al., 2003, 2005; Morales et al., 2017). Quinoa, like cereal crops such as rice, maize, and wheat, is most commonly consumed as seeds (Shi and Gu, 2020; Ma et al., 2021). Quinoa was a major food crop for the Indians, for instance, the Aztec and Inca civilizations. However, the cultivation process was interrupted after the arrival of Spanish immigrants because the conquerors prohibited local quinoa cultivation. Quinoa was rediscovered hundreds of years later, particularly as the twentieth century began, by developed countries due to its comprehensive nutrition. This orphan crop that belonged to poor Andes local farmers suddenly gained attention and became popular worldwide. Compared with cereal crops such as rice, quinoa offers an excellent balance between protein, oil and carbohydrate. The gluten-free starch of quinoa is suitable for celeriac patients (Jacobsen et al., 2003; Vega-Gálvez et al., 2010; Jarvis et al., 2017; Zou et al., 2017). The protein content of quinoa is as high as 15%, with an excellent balance in amino acids, and comparable to cheese and even better than beef (Kozioł, 1992). The lipid composition of quinoa is also superior due to its high concentration of polyunsaturated fatty acids like omega-3 fatty acid and docosahexaenoic acid (DHA), both of which are essential for human physiological demands and fit to a healthy diet. Finally, although the starch content in quinoa seed is relatively low compared to rice and wheat, it can simultaneously fulfil people’s daily energy consumption with a lower glycemic index. Considering its excellent qualities, quinoa grain is a unique plant product covering all the nutrients for human survival and is recommended by the United Nations (FAO) and World Health Organization (WHO) for potential worldwide consumption. Furthermore, the year 2013 was announced as “The International Year of Quinoa” by the United Nations, which implied that the potential of this emerging crop was becoming more widely recognized (Jarvis et al., 2017; Schmöckel et al., 2017).

Quinoa is not only nutritious but also has many excellent agronomic characteristics. Quinoa is highly resistant to various abiotic stresses, including drought, salinity, cold, and soil nutrient defection, which makes it a robust candidate for agricultural development in marginal lands with poor soil conditions (Jacobsen et al., 2005; Razzaghi et al., 2011; Shi and Gu, 2020). Tidal flats and marshlands are potential resources of new farmland worldwide. In such areas, salinity is the primary limiting factor in local agriculture. High salinity can damage cells by causing ionic, osmotic, nutrient, and oxidative stresses, resulting in plants’ growth inhibition and even death (Zhu, 2002; van Zelm et al., 2020; Zhao et al., 2021). As a salt-tolerant crop with high economic and nutrient values, quinoa is undoubtedly an attractive choice. Therefore, salt tolerance is a remarkable agronomy trait of quinoa, and many studies have been conducted, yielding promising results. For example, it has been found that quinoa has a unique tissue (epidermal bladder cell) for salt storage to prevent the somatic cell from salt stress (Zou et al., 2017). Jarvis et al. (2017) completed the first chromosome-level quinoa genome with a size as large as 1.4 Gb, which provides an excellent resource for quinoa molecular and genetic study.

Long non-coding RNAs (LncRNAs) are often recognized as transcripts larger than 200 nt in length but have no apparent protein-coding potential (Kung et al., 2013; Deng et al., 2018). LncRNAs have been discovered to be involved in a variety of biological regulatory processes, and their expression patterns are more tissue-specific than mRNA (Ponting et al., 2009; Rinn and Chang, 2012). According to their relative positions to nearby protein-coding genes in the genome, LncRNAs are generally classified into five types: sense, antisense, bidirectional, intergenic, and intronic (Liu et al., 2012; Lv et al., 2019; Chen et al., 2022). Previous studies have indicated that plant LncRNAs play functional roles in signal pathway transmission and molecular regulation under abiotic stresses such as salt stress. LncRNA973, for example, modulated the expression of a number of salt stress-related genes to positively regulate the response to salt stress in cotton (Zhang et al., 2019). In upland cotton, a competing endogenous RNA of miR160b regulated ARF genes in response to salt via a Long non-coding RNA-lncRNA354 (Zhang et al., 2021). A nucleus-localized drought-induced LncRNA (*DRIR*), which functioned in water transport and ABA signaling, could enhance the tolerance to drought and salt stress in *Arabidopsis* (Qin et al., 2017). Currently, stress-responsive LncRNAs have been identified in many species, such as maize (Lv et al., 2013, 2016; Chen et al., 2022), rice (Zhang et al., 2014), *Arabidopsis* (Liu et al., 2012), pistachio (Jannesar et al., 2020), chickpea (Kumar et al., 2021), and rapeseed (Tan et al., 2020). However, no systematic study on salt-responsive LncRNAs in quinoa has been reported. This study focused on quinoa LncRNAs and their dynamic responses to salt stress. The findings will provide a massive amount of salt-responsive LncRNAs in quinoa and enlighten the potential patterns of LncRNAs incorporation with the coding genes.

## Results

### Transcriptome assembly and long non-coding RNAs identification

To profile the LncRNA transcripts in response to salt stress, we performed the time-series dynamic analysis of RNA-sequencing on quinoa roots exposed to high salinity conditions (Supplementary Table 1). In total, 464 million raw reads were generated, and 430 million clean reads were obtained after cleaning. Approximately 85.30% (367 million) of the clean reads were mapped to the quinoa reference genome and assembled into transcripts. Cleaned data (∼30 Gb) was first mapped to the reference genome, and then alignments were assembled into transcripts in each sample (Supplementary Table 2). Transcripts from all samples were merged to form a unified set of transcripts, which consisted of 188,663 genes and 234,387 transcripts. There were 146,440 transcripts (62.5% of the predicted transcripts) with only one exon. There were 1.24 transcripts per gene and 3.15 exons in a transcript.

**FIGURE 1:**
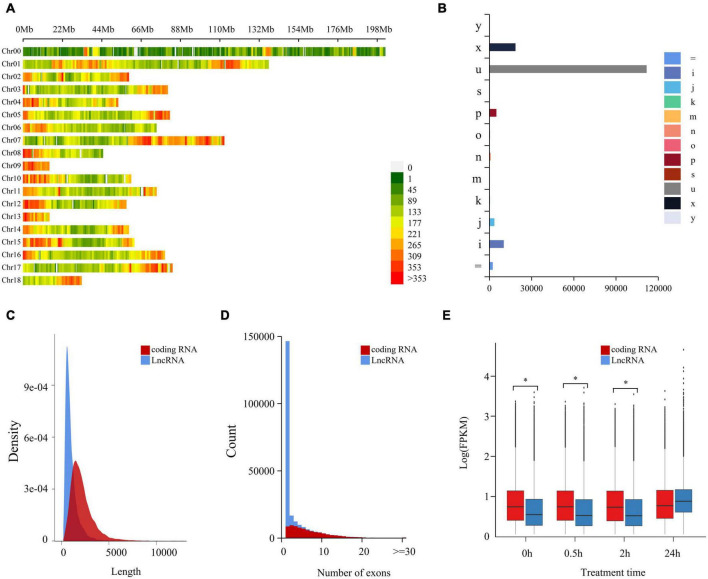
Genome-wide identification and characterization of LncRNA in quinoa root under salt stress. **(A)** Chromosome distribution of LncRNAs in quinoa reference genome. The LncRNAs density was demonstrated by the coloration. Numbers on the right hand of color bar indicated the amount of LncRNAs within 1 Mb window size. Chr: chromosome. **(B)** Annotation classification of LncRNAs based on reference gene set. Class codes were generated by Cuffcompare against quinoa reference gene set (*Chenopodium quinoa* v1.0). Different groups were represented in different colors and marked with characters and symbols. “=”: complete, exact match of intron chain; “i”: fully contained within a reference intron; “j”: multi-exon with at least one junction match; “k”: containment of reference (reverse containment); “m”: retained intron(s), all introns matched or retained; “n”: retained intron(s), not all introns matched or retained; “o”: other same strand overlap with reference exons; “p”: possible polymerase run-on (no actual overlap); “s”: intronic match on the opposite strand (likely a mapping error); “u”: unkown, intergenic; “x”: exonic overlap on the opposite strand; “y”: contains a reference within its intron(s). **(C)** Length distribution of coding RNAs and LncRNAs. **(D)** Exons distribution of coding RNAs and LncRNAs. **(E)** The expression patterns of coding RNAs and LncRNAs at different time points of salt treatment. *p < 0.05 (Student’s t-test).

Coding potential ability was analyzed by CPC2, from which there were 153,751 non-coding LncRNAs identified (Supplementary Table 3). A genome location study showed these LncRNAs were intensely distributed in 18 chromosomes of *C. quinoa*, and the distribution intensity in the two ends of each chromosome was higher than in other parts (Figure 1A). According to the relationship between a transcript and the closest reference transcript, LncRNAs were grouped into different classes represented by characters and symbols by GffCompare software (Figure 1B). “u” was the most abundant type (72.8%), showing that most LncRNAs came from the intergenic region. The second most abundant class was the “x” type (12.1%), followed by “i” (6.7%) and “p”(3.3%). These results indicated that LncRNAs seldom overlapped with reference transcripts. As shown in Figures 1C,D, the length of LncRNAs was generally much shorter than the coding RNAs, and LncRNA had less exon contained than the coding ones. The difference in expression pattern between coding RNAs and LncRNAs was measured statistically using a two-tailed Mann-Whitney *U*-Test (Figure 1E). The expression pattern of LncRNAs was significantly different from that of the coding genes. The overall expression level of LncRNA was significantly lower than that of coding RNAs at 0, 0.5, and 2 h of -salinity treatment (*p* < 2.2e-16). However, at 24 h of treatment, the overall expression level of LncRNAs increased sharply and was more significant than that of coding RNAs. The expression level of coding RNAs was relatively stable within 24 h of treatment. For further validating the reliability of LncRNAs, 50 randomly selected LncRNAs were aligned against Pacbio full-length cDNA datasets from Jarvis et al. (2017). A 47 of 50 LncRNAs were successfully aligned into full-length transcripts. Among them, 46 full-length cDNAs corresponding to LncRNAs were also no coding potential by CPC2 program prediction. These cross-validation results revealed that a set of high-confidence LncRNAs was obtained in the study (Supplementary Table 8).

### Identification of differentially expressed long non-coding RNAs in response to salt stress

**FIGURE 2:**
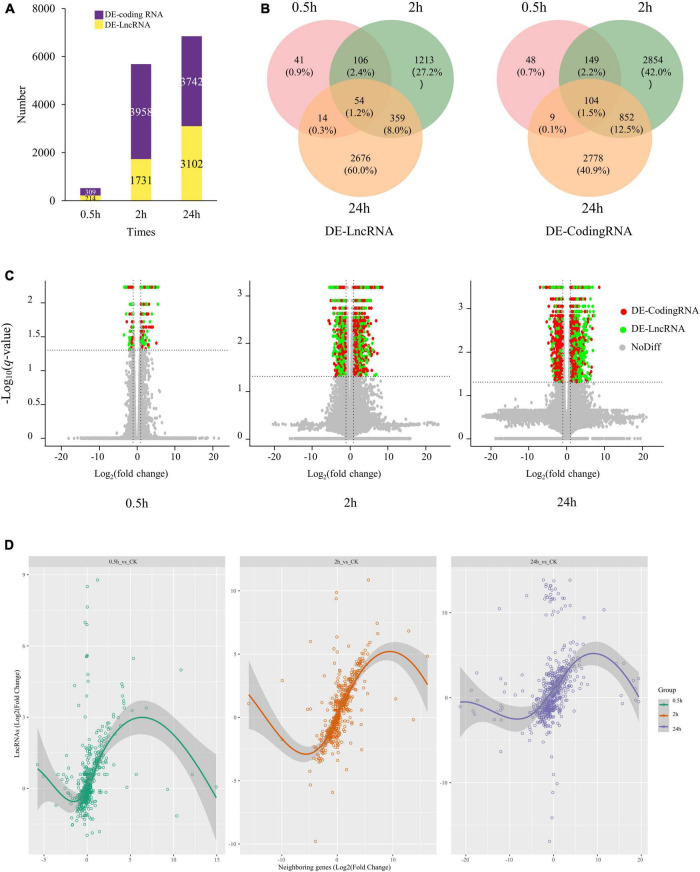
The expression profile of coding RNAs and LncRNAs under salt stress in quinoa roots. **(A)** Number of DE-coding RNAs and DE-LncRNAs identified at 0.5, 2, and 24 h of salt treatment. **(B)** Venn diagrams of DE-coding RNAs and DE-LncRNAs. **(C)** Volcano plots of DE transcripts at 0.5, 2, and 24 h of salt treatment. Red point represented DE-coding RNAs; green point represented DE-LncRNAs; gray point represented non-DE transcripts. **(D)** Correlation plot of salt-responsive LncRNAs and their closest neighboring genes at 0.5, 2, and 24 h under salt stress.

Differentially expressed transcripts, including coding RNAs and LncRNAs, were then identified by DESeq2 (Supplementary Table 4). We identified 4,460 DE-LncRNAs, of which 214, 1,731, and 3,102 were identified at 0.5, 2, and 24 h of treatment (Figure 2A). Only 54 LncRNAs were differentially expressed at all the three-time points, occupying 1.2% of total DE-LncRNAs (4,460) (Figure 2B). Totally 6,791 DE-coding RNAs were also identified, 104 (1.5%) differentially expressed at three-time points (Figure 2B). At 0.5 h of treatment 75% (161) DE-LncRNAs and 77% (237) DE-coding RNAs were upregulated, while at 2 h of treatment 48% (831) DE-LncRNAs and 56% (2,197) DE-coding RNAs were upregulated. At 24 h of treatment, as high as 81% (2,513) DE-LncRNAs were upregulated, whereas only 39% (1,462) DE-coding RNAs were upregulated (Figure 2C and Supplementary Table 2).

Previous studies have suggested lncRNAs may play *cis* regulation role against neighboring genes. To investigate this possibility, we further measured the expression correlation between salt-responsive LncRNAs and their closest neighboring gene in either the 5′ or 3′ direction, yielding a dataset of 1,740 LncRNA-Coding Genes pairs (differentially expressed LncRNAs and their neighboring genes). The correlation analysis showed lncRNAs were strongly and highly significantly correlated with the expression of their closest neighboring gene (*r* = 0.346, *p*-value < 2.2e-16) (Figure 2D). Suggesting that lncRNAs may either be involved in *cis-*acting regulation or are subject to some of the same *cis-*acting regulatory features as their neighboring genes.

### Construction of gene co-expression network and analysis of salinity responsive modules

**FIGURE 3:**
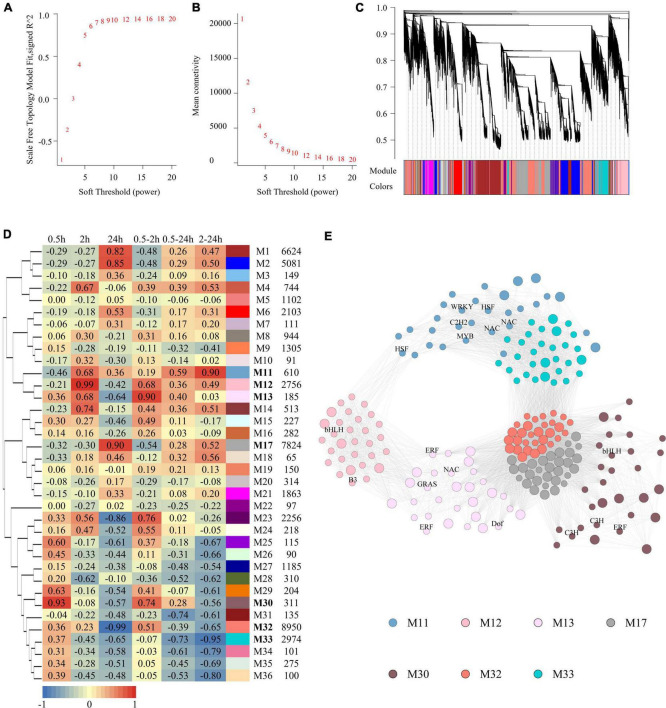
Construction of gene co-expression network and analysis of salt responsive modules. **(A)** The scale-free fit index as a function of the soft-thresholding power. **(B)** The mean connectivity as a function of the soft-thresholding power. **(C)** Clustering dendrogram of genes, with dissimilarity based on topological overlap. Modules were labeled by colors as indicated by the color band underneath the tree. **(D)** Heatmap showing the correlation between modules and salt treatment. Each cell contains the corresponding correlation. The table is color-coded by correlation according to the color legend. The columns on the right of heatmap showed module color, module name and module size in turn. **(E)** Subnetwork of hub genes of salt responsive modules. Modules were represented by different colors as described in Figure 3D. Small circle represented coding RNAs and big circle represent LncRNAs. TFs were labeled.

To infer the potential biological functions of the LncRNAs, a weighted gene co-expression network consisting of both LncRNAs and coding RNAs based on expression profiles was constructed by the WGCNA program (Langfelder and Horvath, 2008). The soft-thresholding power was predicted and defined as 7, as it was the lowest power for which the scale-free topology fit index reached 0.90 (Figures 3A,B). There were finally 36 modules (Figure 3C) generated, and they were named from M1 to M36. The relationship between modules and salinity treatment was calculated, of which seven modules were highly relevant to salinity treatment (Figure 3D). M30 (*r* = 0.93, *p* = 1.57e-05) and M12 (*r* = 0.99, *p* = 9.12e-10) were upregulated significantly at 0.5 and 2 h, respectively; M17 (*r* = 0.90, *p* = 5.48e-05) and M32 (*r* = −0.99, *p* = 4.71e-09) modules upregulated and downregulated at 24 h, respectively; M13 (*r* = 0.90, *p* = 5.53e-05) upregulated at 0.5 and 2 h both; M11 (*r* = 0.90, *p* = 6.35e-05) upregulated at both 2 and 24 h, while M33 (*r* = 0.90, *p* = 6.35e-05) downregulated at the same time. No module was significantly regulated at all the three-time points. The percentage of LncRNAs in salinity-responsive modules ranged from 20 to 40%. The genes with the highest connectivity in each module were selected as hub genes. Totally, 210 hub genes were identified from these seven salinity-responsive modules listed above, which constituted a subnetwork (Figure 3E and Supplementary Table 6). The hub genes of M30 included both TF genes and LncRNAs, and so did M13, M12, and M11 modules. This implied that LncRNAs within these modules might interact with transcript factors and their role in salinity response. However, TF genes were not included in the hub genes of M17 and M32 modules. Instead, more than half of the hub genes were LncRNAs in them. In M17 module as high as 23 LncRNAs were at the hub position (Supplementary Table 6).

### Gene ontology enrichment analysis of salinity-responsive modules

**FIGURE 4:**
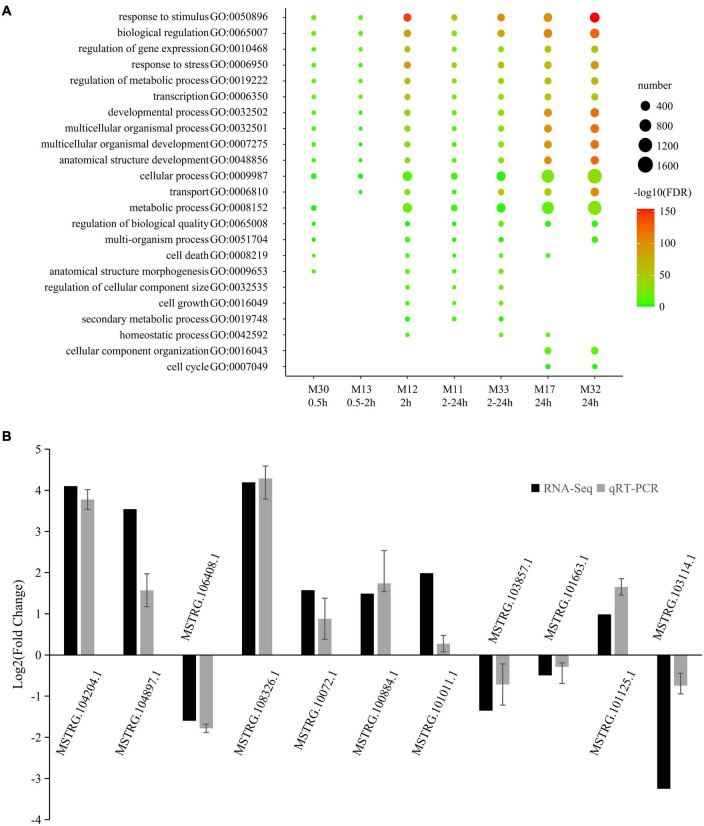
Most representative GO terms of salt responsive modules and qRT-PCR validation. **(A)** Color legend represented –log_10_ FDR. Point size represented number of genes enriched in the GO terms. The rows beneath the bubble chart showed module name, and the response time of each module. **(B)** The qRT-PCR histogram for each locus represents the mean ± standard error (SE) of three independent biological replicates, and the qRT-PCR are compared to fold-change data inferred from RNA-seq data.

Gene ontology analysis of coding RNAs was performed to predict the function of the LncRNAs within the same module (Supplementary Table 5). The most representative GO terms of high-salinity responsive modules are shown in Figure 4A. A large part of the GO terms was enriched in all the seven high-salinity responsive modules. They were related to various critical biological processes, including biological regulation (GO:0065007), regulation of gene expression (GO:0010468), response to stress (GO:0006950), regulation of metabolic process (GO:0019222), transcription (GO:0006350) and developmental process (GO:0032502). Transport (GO:0006810) and metabolic process (GO:0008152) were enriched in six salinity-responsive modules. A small part of the GO terms was enriched in only two or three modules such as cell cycle (GO:0007049). Within these modules, there were several genes directly responding to salt stress. For instance, in M11, a transcript encoding a salt tolerance zinc finger (C2H2 type) which is highly homologous to the *Arabidopsis* gene (AT1G27730.1); in M12, there is a transcript encoding calcineurin B-like protein 1, which is highly homologous to *Arabidopsis* gene (AT4G17615.1), it might function as a positive regulator of salt and drought responses and as a negative regulator of cold response, and mediates the activation of AKT1 by CIPK proteins (CIPK6, CIPK16, and CIPK23) in response to low potassium conditions; In M13, one transcript homologous to *Arabidopsis* gene AT5G12010 was included, which might respond to salt stress and function in ABA-activated signaling pathway.

### Validation of salt-responsive long non-coding RNAs by quantitative real-time PCR analysis

To validate the reliability of salt-responsive LncRNAs, 15 salt-responsive LncRNAs were randomly selected and then subjected the 0 and 2 h salt-treated samples to quantitative real-time PCR (qRT-PCR) to compare expression changes between replicated control and salt-treated. As a result, 11 of 15 salt-responsive LncRNAs were successfully detected and showed a high degree of consistency (*r* = 0.877, *p* = 0.000392) between RNA-seq and qRT-PCR (Figure 4B and Supplementary Table 7).

## Discussion

Because of its remarkable tolerance to soil salt, drought and infertility, high-quality and well-balanced nutrient, quinoa has become popular and well-studied for its unique nature (Jacobsen et al., 2003; Jarvis et al., 2017; Zou et al., 2017). Quinoa is not only a species with high tolerance to salt stress, but also a plant preferring sodium (Wu et al., 2016). Hence, it is acknowledged as halophytic plant species by some studies. Up to date, there have been dozens of studies carried out to dissect the characteristic. It has been demonstrated that the quinoa plant has epidermal bladder cells on its leaves and that these could pump extra sodium chloride into it (Böhm et al., 2018). Sodium cation could be sequestrated in leaf-cell vacuole (Shabala and Mackay, 2011). The cellular potassium retention ability has been enhanced; on the other hand, cellular sodium exclusion and xylem loading in quinoa is also superior (Maughan et al., 2009). However, the salt tolerance trait of quinoa has a complicated underlying mechanism.

Plants have developed a series of physiological functions to alleviate stress or adapt to different environmental changes. The signal pathways and regulation systems lying behind are being dissected more and more. Non-coding RNAs have been illustrated to play important roles in the biological processes. These non-coding RNAs mainly include small non-coding RNA (sncRNA) and long non-coding (LncRNA). miRNA is one of the major parts of sncRNAs, as well as the siRNA; and they mediate gene silencing and hence regulate the expression levels of the genes (Negi et al., 2021). Mul-miR3954, for example, was discovered in *Arabidopsis* to improve the salt-tolerance level of transgenic plants (Gai et al., 2018); miR398b and miR298 could regulate Cu/Zn-*SOD* expression in response to ROS levels induced by salt stress (Feng et al., 2010). TCONS_00009717 is a miRNA found in soybean that may be induced by salt stress, its potential target is cytochrome P450 (Chen et al., 2019). Summarily, LncRNAs were also discovered and found to be involved in response to several abiotic stresses in plant species such as *Arabidopsis thaliana*, *Zea may*, and *Nicotiana tabacum* (Liu et al., 2012; Lv et al., 2013, 2019; Yu et al., 2019; Zhang et al., 2019, 2021; Chen et al., 2022). However, as a remarkable salt tolerant crop, the LncRNAs of quinoa responding to salt stress have not been reported yet.

In the study, we performed a time-course dynamic transcriptome analysis on quinoa under salinity treatment. Under salinity treatments, we extracted 234,387 transcripts from quinoa seedling roots. 153,751 LncRNAs and 51,667 genes were discovered among them. LncRNAs were generally shorter in length than coding RNA, which was likely due to the fact that nearly 90% of LncRNAs had only one exon (Figures 1C,D). In Figure 2B, time-course dynamic transcriptome analysis revealed a total of 4,460 identified DE-LncRNAs and 6,791 DE-coding RNAs and the DE-LncRNAs and DE-coding RNAs showed a time-dependent pattern, respectively. Meanwhile, as shown in Figure 2A, most of DE-LncRNAs were upregulated, which was probably why the overall expression level of LncRNAs increased enormously at 24 h of treatment (Supplementary Table 2).

Previous studies have suggested lncRNAs may play *cis* regulation role against neighboring genes. A dataset of 1,740 LncRNA-Coding Genes pairs (salt-responsive LncRNAs and their closest neighboring gene in either the 5′ or 3′ direction) was generated. Pearson correlation analysis showed highly significantly correlation level (*r* = 0.346, *p*-value < 2.2e-16) (Figure 2D), which illustrated that LncRNAs may play a *cis-*acting regulation role on their neighboring genes under salt stress.

The DE-LncRNAs and DE-coding RNAs were further examined, yielding 36 weighted gene co-expression network modules, seven of which showed responses to salinity stress. Highly connected hub genes in a module most likely played important roles in the same biological processes. The hub genes from each of the seven modules were shown as a subnetwork in Figure 3E. Some DE-coding RNAs are also classified as transcription factors (TF). For example, in module 11, one transcript encodes a homologous of *Arabidopsis* gene (ATG27730.1), which encodes a zinc finger TF involved in salt tolerance. It is also a hub gene in that module with high connectivity with others. In *Arabidopsis*, the homologous gene could respond to various kinds of stresses such as salt, cold, drought and oxidative. It is closely associated with the signal pathway of stress tolerance in plants. In modules 17 and 32, there were no TFs involved, which indicated that some LncRNAs might also work independently of TFs in response to salinity stress in quinoa. Another 48 TFs belonging to coding RNAs and 62 LncRNAs in this module implied that LncRNAs within these modules might interact with transcript factors and play a hub role in salinity response.

## Conclusion

As a well-known salt-tolerant crop, few reports have identified long non-coding RNAs under salt stress in quinoa. Hence, we provided a bulk of LncRNAs in quinoa roots on a large scale and identified those induced or suppressed by salt treatment. Furthermore, we also predicted the potential gene-expression modules in which the LncRNAs might be included and function together with genes. Hopefully, these findings will serve as a dataset resource for further research on quinoa salt tolerance and provide a reference for quinoa breeding work.

## Materials and methods

### Plant material and salt treatment

*Chenopodium quinoa* cultivar QQ056 were acquired from the USDA-ARS National Plant Germplasm System (NPGS) with permission for scientific research. Detailed information on the variety could be found at npgsweb.ars-grin.gov (accession: PI 584524). The experiments were performed in a phytotron at 28°C under 16 h/8 h (day/night) photoperiod. Seeds of quinoa were surface sterilized, germinated for 7 days, and then moved into a half-strength Hoagland solution. Seedlings of 28-day-old were transferred to half-strength MS with 300 mM NaCl for salt treatment. Roots of quinoa were gathered at 0, 0.5, 2, and 24 h of treatment and stored at –80°C for further investigation. Three biological repeats were used at each time point.

### Library preparation and RNA-seq sequencing

Total RNA was isolated, purified and concentrated with an RNAprep Pure Plant Kit (Tiangen, China). A Thermo 2000 Bioanalyzer evaluated the concentration and quality of RNA with an RNA NanoDrop (Thermo Scientific, United States). cDNA libraries were conducted using TruSeq mRNA Sample Prep Kit (Illumina, United States). RNA sequencing (RNA-seq) was performed on the Illumina NovaSeq 6000 platform (Illumina, United States) at Annoroad Gene Technology (Beijing) Co., Ltd., China.

### RNA-seq data analysis

Raw data were trimmed with low-quality bases and short reads (< 50 bp) using Fastp v0.23.2 (Chen et al., 2018). Cleaned reads were then mapped into the quinoa reference genome (Jarvis et al., 2017) by splice-aware alignment method using STAR v2.7.10a with two-pass mode (Dobin et al., 2013). The mapped reads of each sample were separately assembled by the reference annotation-based transcript (RABT) assembly algorithm and then combined with known transcript annotation (Jarvis et al., 2017) into an updated GTF file using StringTie v2.2.1 (Pertea et al., 2016). Finally, the abundance of transcripts was quantified and normalized with HTseq (Putri et al., 2022) and DESeq2 (Love et al., 2014). The transcripts with fragments per kilobase of transcript per million fragments mapped (FPKM) < 1 in more than three samples were excepted for downstream analysis.

### Coding potential prediction of long non-coding RNAs

Computational prediction of quinoa LncRNAs was followed as described by Lv et al. (2019) with some custom modification. Sequences of transcripts were firstly retrieved using Gffread v0.12.2 (Pertea and Pertea, 2020). Then transcripts were evaluated in their coding potential using CPC v2.0 (Kang et al., 2017). Default parameters were used. Finally, non-coding transcripts longer than 200 bp were considered as LncRNAs. The updated GTF file was also compared with the reference GTF file using gffCompare (Pertea et al., 2016) to generate class codes representing the position information between the updated transcript and the closest reference transcript.

### Differential expression analysis

For differential expression analysis, we compared every time point (0.5, 2, and 24 h of treatment) with CK (0 h) using DESeq2 (Trapnell et al., 2010) based on the negative binomial distribution. Differentially expressed should be fulfilled the following criteria: (I) fold change > = 1; (II) adjusted *p* value < 0.05.

### Gene co-expression network analysis

A weighted co-expression network was further constructed for linking coding RNAs and LncRNAs using the WGCNA program (Zhang and Horvath, 2005; Langfelder and Horvath, 2008). The correlation between module eigengenes and salt treatment was then calculated. Modules that showed a significant correlation (*| r*| > 0.9, *p* < 0.001) with a specific time of treatment were recognized as salt-responsive modules (Xue et al., 2013). The hub genes of each module were worked out based on the Topological overlap matrix (TOM).

### Gene ontology enrichment analysis

Gene Ontology (GO) enrichment analysis was performed with agriGO v2.0 toolkit (Tian et al., 2017). Fisher’s exact test was applied for the enrichment analysis, and the false discovery rate (FDR) was assessed using the Yekutieli method. GO terms with an FDR less than 0.05 were considered significant.

### Quantitative real-time PCR validation

To validate the reliability and accuracy of the LncRNA analysis, ten salt-responsive LncRNAs were chosen and measured by quantitative real-time PCR (qRT-PCR). The first-strand cDNA of 0 and 2 h samples were synthesized using the PrimeScript TM RT-PCR Kit (Takara, Japan). We conducted the qRT-PCR on an ABI StepOnePlus Real-Time PCR System (Applied Biosystems, CA, United States) with FastStart Universal SYBR Green Master (Roche, Germany) according to the manufacturer’s protocol. The primers were designed using Primer Premier v.5.0 software (Premier Biosoft International, CA, United States). CqEF1a (AUR62020767) was used as an internal standard to normalize the relative expression level and determine expression values based on the 2^–ΔΔCt^ method. The primers for qRT-PCR were presented in Supplementary Table 7.

## Data availability statement

The datasets presented in this study were deposited in National Center for Biotechnology Information (NCBI) BioProject database under accession number: PRJNA636120. All data can be found in the article/Supplementary material.

## Author contributions

YL and XC conceived and designed the experiments. CL, BH, PS, JX, HG, BP, JC, and FH participated in experiments and data analyses. CL, BH, and PS wrote the manuscript with inputs and guidance from YL. All authors have read and approved the final manuscript.
